# Perceived Discrimination and Subjective Well-being in Chinese Migrant Adolescents: Collective and Personal Self-esteem As Mediators

**DOI:** 10.3389/fpsyg.2017.01213

**Published:** 2017-07-17

**Authors:** Xuji Jia, Xia Liu, Baoguo Shi

**Affiliations:** ^1^Key Research Base of Humanities and Social Sciences of Ministry of Education, Academy of Psychology and Behavior, Tianjin Normal University Tianjin, China; ^2^Center of Collaborative Innovation for Assessment and Promotion of Mental Health Tianjin, China; ^3^Institute of Developmental Psychology, Beijing Normal University Beijing, China; ^4^Department of Psychology, Capital Normal University Beijing, China

**Keywords:** perceived discrimination, collective self-esteem, personal self-esteem, subjective well-being, Chinese migrant adolescents

## Abstract

This study aimed to examine whether collective and personal self-esteem serve as mediators in the relationship between perceived discrimination and subjective well-being among Chinese rural-to-urban migrant adolescents. Six hundred and ninety-two adolescents completed a perceived discrimination scale, a collective self-esteem scale, a personal self-esteem scale, and a subjective well-being scale. Structural equation modeling was used to test the mediation hypothesis. The analysis indicated that both collective and personal self-esteem partially mediated the relationship between perceived discrimination and subjective well-being. The final model also revealed a significant path from perceived discrimination through collective and personal self-esteem to subjective well-being. These findings contribute to the understanding of the complicated relationships among perceived discrimination, collective and personal self-esteem, and subjective well-being. The findings suggest that collective and personal self-esteem are possible targets for interventions aimed at improving subjective well-being. Programs to nurture both the personal and collective self-esteem of migrant adolescents may help to weaken the negative relationships between perceived discrimination and subjective well-being.

## Introduction

Based on the Sixth National Population Census of China of 2010, more than 236 million people in China have migrated from rural to urban areas in search of better living conditions; 35.81 million of them are children under the age of 18 (National Committee on Family Planning, 2013). These migrant children who accompany their parents cannot enjoy rights equal to local children in the cities because of the household registration system (Hukou). In China, there are dual Hukou for urban and rural areas. Urban residents obtain city Hukou and rural residents obtain rural Hukou. The Hukou was designed to control rural–urban mobility for economic and political purposes, but it also permits residents access to a variety of amenities provided by their city. Chinese rural-to-urban migrants in cities do not have a local household registration; they are considered temporary residents and are not granted equal access to education, medical care, and other social services in the cities where they live.

In recent years, a number of studies in China have compared migrant children’s emotional and behavioral problems with those of urban children (Shen, 2009; Yin and Liu, 2013). However, much of this research has focused on negative outcomes in migrant children (e.g., depression, anxiety, and behavioral problems) and there is a dearth of studies on positive developmental outcomes. Subjective well-being (SWB), an important area of research in positive psychology, reflects individuals’ cognitive and affective evaluations of the quality of their lives. SWB is comprised of both cognitive and affective components (Diener and Diener, 1995). The cognitive component refers to life satisfaction that results from a subjective evaluation of overall quality of life, and the affective component refers to levels of positive affect and negative affect, which indicate SWB. One study that examined SWB in Chinese migrant children compared with urban children in Beijing city found that migrant children reported lower levels of general well-being, life satisfaction, and positive affect, along with higher levels of negative affect (Wang and Zou, 2010). These differences suggest that further studies should focus on the identification of factors related to SWB in Chinese migrant children, especially for adolescents who are in a stage of life characterized by rapid changes, with specific biological, psychological, and sociological aspects. However, most studies on SWB have examined adults. Research on adolescents’ SWB is still in its infancy (Ronen and Seeman, 2007). Recent studies have found that adolescents present lower levels of SWB with increased age (Ronen et al., 2016). For these reasons, it is critical to examine the mechanisms by which psychological factors may predict SWB, which would help us identify potential opportunities to develop effective intervention programs designed to improve Chinese migrant adolescents’ positive development.

When migrant adolescents move from rural to urban areas, they must settle into a new environment and potentially are faced with a variety of stressors and challenges. One of the most important stressors in rural-to-urban migration is discrimination and perceived discrimination (Shen, 2009). Perceived discrimination is a type of social stress experienced commonly by low-status groups because of their group membership (e.g., ethnicity, and—here—Hukou), which refers to the subjective experience of being treated unfairly (Flores et al., 2008). Previous studies have found that adolescents are particularly sensitive to the level of emotional stress and crisis (Dahl, 2001), and that their experience of discrimination increases (Fisher et al., 2000). This raises the question of how perceived discrimination is linked with adolescents’ SWB during this rapidly changing developmental period.

Chinese rural-to-urban migrant adolescents tend to experience discrimination. In one study, 75.7% of migrant children (including adolescents) reported experiencing discrimination–such as sarcasm or insults–in their daily lives (Lei, 2004). Several studies have documented the negative links between perceived discrimination and migrant adolescents’ psychological adaptation, including feelings of loneliness, symptoms of depression and anxiety, low levels of self-esteem, and low levels of SWB (Lin et al., 2009). Although research has established a relationship between perceived discrimination and adjustment, the relationship among perceived discrimination, specific mediators—especially self-esteem—and Chinese migrant adolescents’ SWB, have not been examined.

Self-esteem is strongly related to several measures of well-being or adjustment, such as life satisfaction (Douglass and Duffy, 2014), positive and negative affect (Shen, 2009), happiness (Simsek, 2011), loneliness (Lin et al., 2009), depression and anxiety (Cassidy et al., 2004; Fischer and Holz, 2007). Although Heine et al. (1999) found that self-esteem needs are less pressing in Japan than in North America and suggested that self-esteem may be a uniquely Western phenomenon, recent study using meta-analysis concluded that the need for high self-esteem is not unique to Western cultures but is also experienced in China (Cai et al., 2009). Moreover, a great deal of research has found that self-esteem is significantly associated with Chinese participants’ psychological well-being (Cai et al., 2009, 2011), and high self-esteem serves to buffer the relationships between stressful events and adolescent depression (Deng et al., 2013).

Luhtanen and Crocker (1992) distinguished two components of self-esteem: personal self-esteem and collective self-esteem. The distinction between personal and collective self-esteem reflects the distinction between personal identity (i.e., the self as a unique individual) and social identity (i.e., the self as a group member, Tajfel and Turner, 1986). Personal self-esteem refers to feelings of self-worth and self-respect and is related to one’s understanding of themselves as an individual, whereas collective self-esteem refers to feelings of self-worth and self-respect related to one’s self-concept as a member of a social group (Crocker et al., 1994). Research has shown the distinct and unique effects of personal and collective self-esteem on psychological adjustment (Cassidy et al., 2004). For example, Crocker et al. (1994) found that collective self-esteem predicted psychological adjustment beyond the contributions that could be attributed to personal self-esteem. Other work with Chinese adolescents indicated that personal self-esteem could significantly predict life satisfaction, positive affect, and negative affect, while collective self-esteem only predicted positive affect (Deng et al., 2015). Thus, it is important to consider both personal self-esteem and collective self-esteem to develop a comprehensive understanding of mental health outcomes in minority groups (e.g., Chinese migrant adolescents).

Because self-esteem is an important aspect of psychological well-being, it is not surprising that it has received substantial attention in the literature as a possible mediator of the association between stress and adjustment. According to the stress-and-coping model (Lazarus and Foulkman, 1984), when individuals appraise a negative event (e.g., discrimination) as stressful, they perceive their self-image to be threatened. This threat may have significant prediction for an individual’s self-esteem or self-evaluation, which may, in turn, directly link with their levels of psychological well-being. There is also evidence suggesting that self-esteem may mediate or explain the perceived discrimination–adjustment connection. For example, Umaña-Taylor and Updegraff (2007) found that personal self-esteem partially mediated the relation between perceived discrimination and depressive symptoms in adolescents. Cassidy et al. (2004) found that both personal and collective self-esteem mediated the discrimination–distress relationship among young ethnic minority men. Previous research tended to support the hypothesis that self-esteem, whether personal or collective, mediates the link between perceived discrimination and adjustment outcomes. One of the main limitations of previous studies, however, is that they have focused on personal self-esteem or have examined the mediating roles of personal and collective self-esteem separately. Very little research has examined the relationships among perceived discrimination, personal and collective self-esteem, and psychological distress (Fischer and Holz, 2007). Fischer and Holz (2007) found that perceived sexist discrimination partially predicted lower personal self-esteem through the indirect link between perceived sexist discrimination and collective self-esteem, linking with psychological distress. Consequently, additional research examining the concurrent mediating roles of personal and collective self-esteem would contribute to a richer understanding of the link between perceived discrimination and well-being.

Additionally, for members of low-status groups, the permeability of group boundaries (i.e., opportunities for individual upward mobility) is linked with how they respond to discrimination against their group (Garstka et al., 2004). For example, according to the rejection–identification model (Branscombe et al., 1999), perceptions of discrimination have a positive indirect association with well-being, mediated by increased in-group identification. However, Garstka et al. (2004) examined the roles of perceived age discrimination in the relationships between well-being and group identification, and found that this process model was only confirmed among older adults because their low status group membership is permanent but not confirmed among young adults whose low status could be temporary. Previous studies that examined whether or not self-esteem mediates the relationship between perceived discrimination and well-being primarily have focused on group membership that is relatively enduring and permanent (e.g., gender, race). For Chinese migrant adolescents, their membership (i.e., Hukou) in the migrant group depends entirely on their current residence location, which could be transitory^1^. They become migrant adolescents upon their family’s relocation from a rural area to an urban area. After middle school, migrant adolescents have few educational opportunities in the city and must return to their rural hometowns to take the high-school entrance examination^2^ (Shen, 2009). Thus, the permeability of these adolescents’ group membership is remarkable compared with other low-status groups. This greater individual mobility should be related to psychological responses to perception of discrimination (Garstka et al., 2004). However, little research has been conducted on the relationship between perceived discrimination, collective and personal self-esteem, and SWB in low-status groups for whom membership is transitory (e.g., Chinese migrant adolescents). The present study was designed address that gap.

**FIGURE 1:**
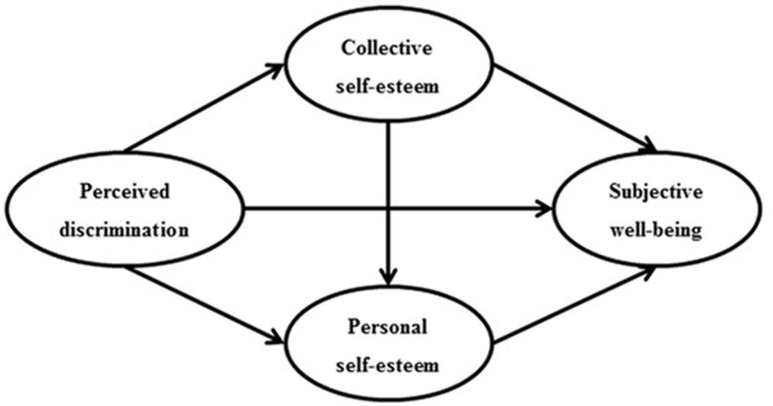
Hypothesized model. Unidirectional arrows indicate the path of one variable on another.

In summary, the purpose of this study was to examine the mediating roles of both personal and collective self-esteem on the relationship between perceived discrimination and SWB (i.e., life satisfaction, positive and negative affect) among Chinese migrant adolescents. Consistent with previous literature (Cassidy et al., 2004; Fischer and Holz, 2007), we hypothesized that the relationship between perceived discrimination and SWB would be at least partially mediated by personal and collective self-esteem. Previous research has indicated that collective self-esteem was indirectly linked to perceived sexist discrimination and personal self-esteem (Fischer and Holz, 2007), and that a personal sense of self-worth is dependent upon collective sources of esteem (Simsek, 2011). Therefore, we also expected that collective self-esteem would mediate the relationship between perceived discrimination of migrant children and personal self-esteem. In turn, personal self-esteem would prove to be a mediator between collective self-esteem and SWB. Specifically, we assumed a chain of mediation that perceived discrimination would predict collective self-esteem, which may have an association with personal self-esteem and in turn, predict Chinese migrant adolescents’ SWB. The expected relationships among the study variables are depicted in **Figure 1**.

## Materials and Methods

### Participants

Six hundred and ninety-two migrant students (351 males and 341 females) were recruited from eight schools in Beijing. The principals of four migrant schools (where 100% of the students were migrant children) and four public schools (where 58.2% were migrant children) in Beijing agreed to have their schools participate in the study. Public schools usually provide better access to educational resources and high quality education, in contrast to migrant schools, which usually are poorly constructed and provide a lower quality education. All participants were rural-to-urban migrant adolescents. None of them possessed a Beijing Hukou. The average age was 13.37 years (*SD* = 1.48). 230 (33.2%) were from public school, and 462 (66.8%) were from migrant school. The average length of residence in the city was 4.22 years (*SD* = 1.49). The school sample covered 179 (25.9%) students in the fifth grade, 176 (25.4%) in the sixth grade, 183 (26.4%) in the seventh grade, and 154 (22.3%) in the eighth grade. All of the participants belonged to the Han ethnic group, which is the predominant ethnic group in China and accounts for more than 90% of the population.

### Measures

#### Perceived Discrimination

Perceived discrimination was measured with the Perceived Discrimination Scale for Chinese migrant children (Shen and Chen, 2014). The scale consists of 20 items and adolescents rate items on a 5-point Likert scale ranging from “strongly disagree” to “strongly agree.” The scale measures the extent to which individuals perceive discrimination against migrant children (e.g., “Children in Beijing are unwilling to study together with me”). Higher average scores reflect more perceived discrimination. This instrument has been tested on samples of Chinese migrant adolescents and shows good reliability and validity. The correlation is 0.59 (*p* < 0.01) between the score of perceived discrimination and score of discrimination events, indicating a good criterion validity (Shen and Chen, 2014). In this study, the Cronbach’s alpha coefficient was 0.87.

#### Personal Self-esteem

The Rosenberg (1965) Self-Esteem Scale was used to measure adolescents’ personal self-esteem. Using a 5-point Likert scale, adolescents rated their agreement with 10 items, such as “I am able to do things as well as most other people.” Scores on the 10 items were averaged, with higher scores representing greater personal self-esteem. The scale was widely used to assess the level of personal self-esteem in Chinese samples (Chen et al., 2015; Li and Yin, 2015). Cronbach’s alpha coefficient for the scale was 0.78 in the present study.

#### Collective Self-esteem

A modified version of Luhtanen and Crocker’s (1992) Collective Self-Esteem (CSE) Scale was used to assess how adolescents evaluate the migrant group to which they belong. The original wording of the items with a back-translation was modified in the Chinese version to reflect CSE with regard to one’s group. The modified scale consisted of three subscales (with four items each): Membership CSE (e.g., I am a worthy member of the children’s group from outside Beijing), Public CSE (e.g., In general, others respect the children’s group from outside Beijing), and Private CSE (e.g., Overall, I often feel that the children’s group from outside Beijing is not worthwhile). Adolescents responded to items using a 6-point scale ranging from 1 (strongly disagree) to 6 (strongly agree). In a study about migrant children (Peng et al., 2011), confirmatory factor analysis of the modified scale indicated that the model’s fit indices are GFI = 0.84, CFI = 0.701, RMSEA = 0.101. This same scale has been used to assess the level of collective self-esteem for different groups (Peng et al., 2012; Jia et al., 2015). In our study, Cronbach’s alpha coefficient for the three subscales and total scale were, respectively, 0.55, 0.59, 0.60, and 0.80.

#### Subjective Well-being

We assessed both cognitive and affective dimensions of SWB. The Life Satisfaction Scale (Huebner, 1994) was used to measure the cognitive dimension of SWB. The scale contains seven items rated on a 5-point Likert scale ranging from 1 (strongly disagree) to 5 (strongly agree). The seven items reflect satisfaction with life as a whole (e.g., There are many things that I can do well). The mean scores for all items were used in the analyses. The instrument was adopted to assess the level of life satisfaction among Chinese adolescents (Hou et al., 2009; Liu et al., 2013). The Cronbach’s alpha coefficient was 0.70.

The affective dimension of SWB was evaluated using the Positive/Negative Affect Scale revised by Chen and Zhang (2004). The revised scale was based on Bradburn’s (1969) Affect Balance Scale. This scale consists of eight positive affect items (e.g., During the past few weeks, did you feel particularly interested in something?) and six negative affect items (e.g., During the past few weeks, did you feel depressed or unhappy). Adolescents were asked to rate the emotions they experienced during the past few weeks. Each item was rated on a 4-point scale from 1 (none) to 4 (often). In Chen’s study, confirmatory factor analysis of the scale indicated that the model fit indices were χ^2^ = 594.07, df = 76, GFI = 0.91, CFI = 0.94, RMSEA = 0.06, and SRMR = 0.08. In our study, Cronbach’s alpha for positive affect and negative affect was 0.76 and 0.74, respectively.

### Procedure

Data collection took place from September to December, 2014. Participants were recruited from eight schools in Beijing. They completed the survey in their classrooms during school hours using a paper–pencil format. This study was approved by the Research Ethics Committee of Beijing Normal University, and complied with the Declaration of Helsinki involving human subjects. Prior to survey administration, the researchers obtained consent forms signed by students and their parents. Students were informed that participation was voluntary and that they could either refuse to complete the surveys or withdraw from the study at any time. Half of participants were asked to answer questions that included demographic information, the level of perceived discrimination, personal and collective self-esteem, then report on life satisfaction and measures regarding positive/negative affect. The other half of the participants were instructed to answer demographic information, life satisfaction and positive/negative affect measures, personal and collective self-esteem, and then report on the level of perceived discrimination. The order wasn’t associated with the findings regarding perceived discrimination. The survey was guided by trained research assistants.

### Data Analysis

We used structural equation modeling (SEM) to test our mediation hypothesis. There were four latent variables in the hypothesized model: perceived discrimination, collective self-esteem, personal self-esteem, and SWB. Collective self-esteem was indicated by membership CSE, public CSE, and private CSE. SWB was indicated by life satisfaction and affect.

Perceived discrimination and personal self-esteem were indicated by three item parcels, respectively, which were created using the item parceling method. We used item parceling to control for inflated measurement errors caused by multiple items on perceived discrimination and personal self-esteem. The item-to-construct balance technique (Little et al., 2002) was used to form three parcels per latent variable. Because of the unequal number of items in each parcel, the average scores of the items were used.

Structural equation modeling was conducted using Mplus 7.0 software (Muthén and Muthén, 2010) using the maximum likelihood estimation method. The model fit was evaluated with the chi-square statistic (χ^2^), the root mean square error of approximation (RMSEA) with 90% confidence intervals (CI), the comparative fit index (CFI), the Tucker–Lewis index (TLI), and the standardized root mean squared residual (SRMR). According to the recommendations by Hu and Bentler (1999), RMSEA values ≤ 0.08, CFI values ≥ 0.90, TLI values ≥ 0.90, and SRMR values ≤ 0.08 are considered adequate and indicative of good fit. To ascertain the model that best fit our data, we established several alternative models. The χ^2^ difference test was used to compare the hypothesized and alternative models.

When the final model was selected, bias-corrected bootstrapping, a non-parametric resampling procedure, was used to further examine the significance of the mediating roles. Bootstrapping has greater statistical power to estimate indirect effect than traditional mediation analysis (MacKinnon et al., 2004). If none of the 95% CI were zero, the indirect effect was regarded as statistically significant. In our study, 1,000 bootstrapping samples were generated using random sampling with replacement from the data set.

## Results

### Descriptive Statistics

Correlations, means, and standard deviations for the major variables are presented in **Table 1**. As shown, perceived discrimination was negatively correlated with different dimensions of collective self-esteem (i.e., membership CSE, private CSE, and public CSE), personal self-esteem, life satisfaction, and negative affect. There were positive correlations among collective self-esteem, personal self-esteem, life satisfaction, and positive affect, whereas negative associations were found between negative affect and the other variables (collective self-esteem, personal self-esteem, and life satisfaction). Age was positively correlated with perceived discrimination and negative affect, and negatively associated with the other variables. Moreover, male children reported more perceived discrimination and less collective self-esteem than did female children. Compared with children in public schools, children in migrant schools exhibited higher scores for perceived discrimination and negative affect, lower scores for self-esteem (i.e., membership CSE and personal self-esteem) and life satisfaction. Additionally, children with more years in the city of residence were found to be associated with lower scores for perceived discrimination and negative affect, and higher scores for self-esteem and life satisfaction. Because the relationships between demographic variables (gender, age, school type, and length in city) and major variables were not a major concern in the present research, we regarded the demographic variables as covariates in the subsequent analysis.

**Table 1:** Correlations, means, and standard deviations for the variables (*N* = 692).

Variables	1	2	3	4	5	6	7	8	9	10	11	12
(1) Gender	1											
(2) School type	0.04	1										
(3) Age	0.07	0.02	1									
(4) Length residence in city	0.03	-0.28***	-0.06	1								
(5) Perceived discrimination	0.11**	0.34***	0.20***	-0.11**	1							
(6) Membership CSE	-0.13**	-0.12***	-0.13***	0.10**	-0.33***	1						
(7) Private CSE	-0.08*	-0.05	-0.12**	0.06	-0.33***	0.55***	1					
(8) Public CSE	-0.12***	-0.05	-0.11**	0.08*	-0.41***	0.60***	0.58***	1				
(9) Personal self-esteem	-0.04	-0.21***	-0.08*	0.15***	-0.37***	0.48***	0.38***	0.42***	1			
(10) Life satisfaction	0.05	-0.20***	-0.16***	0.14***	-0.30***	0.22***	0.26***	0.25***	0.32***	1		
(11) Positive affect	-0.01	-0.18***	-0.06	0.17***	-0.29***	0.35***	0.32***	0.33***	0.52***	0.39***	1	
(12) Negative affect	0.02	0.15***	0.23***	-0.09*	0.43***	-0.32	-0.31***	-0.36***	-0.45***	-0.36***	-0.31***	1
Mean	–	–	13.37	4.22	2.42	4.63	4.90	4.57	4.70	3.06	3.09	2.22
SD	–	–	1.48	1.49	0.67	0.93	0.92	0.98	0.80	0.69	0.52	0.62


*CSE, Collective self-esteem; Gender: 0, Female, 1, Male. School type: 0, Public school, 1, Migrant school. ^∗^*p* < 0.05, ^∗∗^*p* < 0.01, ^∗∗∗^*p* < 0.001.*

### Structural Equation Model Testing

The mediation hypotheses for SWB were tested by examining the fit of a series of structural equation models to the data. **Table 2** shows the model fit indices of the hypothesized model and several alternative models. The fit indices indicated that the hypothesized model showed a good fit to the data: χ^2^ = 238.28, df = 82, RMSEA = 0.052 (90% CI for RMSEA = 0.045–0.060), CFI = 0.96, TLI = 0.94, SRMR = 0.03.

**Table 2:** Fit indices of the hypothesized model and alternative models.

Models	χ^2^	df	RMSEA [90% CI]	CFI	TLI	SRMR
Hypothesized model	238.28^∗∗∗^	82	0.052 [0.045, 0.060]	0.96	0.94	0.03
Alternative model 1	253.64^∗∗∗^	83	0.055 [0.047, 0.062]	0.96	0.94	0.04
Alternative model 2	247.56^∗∗∗^	83	0.054 [0.046, 0.061]	0.96	0.94	0.04
Alternative model 3	247.22^∗∗∗^	83	0.053 [0.046, 0.061]	0.96	0.94	0.04
Alternative model 4	346.60^∗∗∗^	83	0.068 [0.060, 0.075]	0.93	0.91	0.07
Alternative model 5	238.28^∗∗∗^	82	0.052 [0.045, 0.060]	0.96	0.94	0.03


**N* = 692. RMSEA, root mean square error of approximation; CI, confidence intervals; CFI, comparative fit index; TLI, Tucker–Lewis index; SRMR, standardized root mean squared residual. ^∗∗∗^*p* < 0.001.*

In alternative model 1, the direct path from perceived discrimination to SWB was constrained to zero, testing the partially mediating roles of collective and personal self-esteem on the relationship between perceived discrimination and SWB. The χ^2^ difference test [Δχ^2^(1) = 15.36, *p* < 0.001] between the hypothesized model and alternative model 1 indicated that alternative model 1 had a significantly worse fit to the data than the hypothesized model, indicating that the direct path from perceived discrimination to SWB should be retained in the model.

In alternative model 2, we set the direct path from perceived discrimination to personal self-esteem to zero, testing the partially mediating role of collective self-esteem on the relationship between perceived discrimination and personal self-esteem. The χ^2^ difference test [Δχ^2^(1) = 9.28, *p* < 0.001] between the hypothesized model and alternative model 2 was significant, suggesting that constraining this path significantly reduced the model fit and that the path from perceived discrimination to personal self-esteem should not be omitted.

The same method was used for the direct path from collective self-esteem to SWB to examine the mediating role of personal self-esteem on the relationship between collective self-esteem and SWB. The χ^2^ difference test [Δχ^2^(1) = 8.94, *p* < 0.001] demonstrated that alternative model 3 had a significantly worse fit to the data than the hypothesized model, indicating that the direct path from collective self-esteem to SWB should be retained in the model.

In alternative model 4, the direct path from collective self-esteem to personal self-esteem was constrained to zero, testing whether collective self-esteem and personal self-esteem were independent in its roles on the relationship between perceived discrimination and SWB. The χ^2^ difference test [Δχ^2^(1) = 108.32, *p* < 0.001] between the hypothesized model and alternative model 4 indicated that alternative model 4 had a significantly worse fit to the data than the hypothesized model.

**FIGURE 2:**
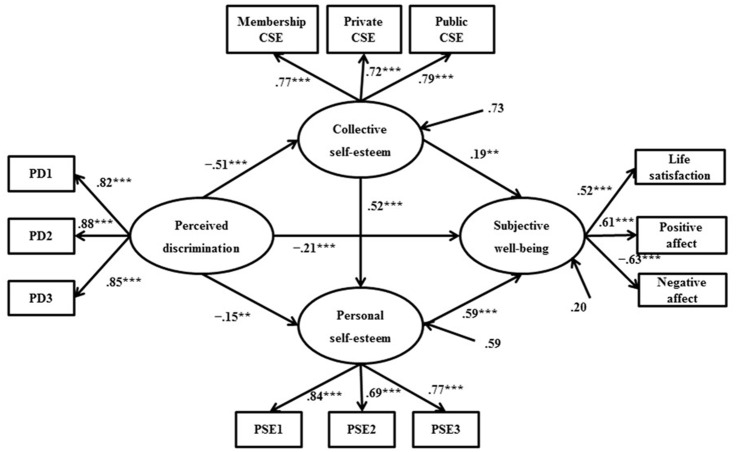
Standardized path coefficients in the final model (*N* = 692). PD1–PD3 = three item parcels from the Perceived Discrimination Scale. CSE, Collective Self-Esteem. PSE1–PSE3 equal three item parcels from the Personal Self-Esteem Scale. ^∗∗^*p* < 0.01, ^∗∗∗^*p* < 0.001.

In alternative model 5, we established a model where SWB is the mediator and collective self-esteem and personal self-esteem are the outcomes. Results showed that the model fit indices are equivalent to the hypothesized model. However, the direct path from perceived discrimination to personal self-esteem is non-statistically significant (β = 0.09, *p* > 0.05). Due to the close relationship identified between perceived discrimination and personal self-esteem in previous studies (Umaña-Taylor and Updegraff, 2007; Liu et al., 2013), the hypothesized model was regarded to be the final model. The parameter estimates of the final model are presented in **Figure 2**.

To further test the significance of the mediating roles, a bias-corrected bootstrap estimation procedure was employed. **Table 3** presents a summary of this analysis, displaying the mediating associations of the study variables. As indicated, the partially indirect effect of perceived discrimination on SWB via collective self-esteem and personal self-esteem was significant (β = -0.16, *p* < 0.001; 95% CI [-0.21, -0.10]; partial mediation: 29.09% of the total effect). The partially indirect effect of perceived discrimination on SWB through collective self-esteem alone also was significant (β = -0.10, *p* < 0.05; 95% CI [-0.17, -0.03]; partial mediation: 18.18% of the total effect). A similar partially indirect effect of personal self-esteem was found for the relationship between perceived discrimination and SWB (β = -0.09, *p* < 0.05; 95% CI [-0.15, -0.03]; partial mediation: 16.36% of the total effect). Perceived discrimination wielded a significant indirect prediction on personal self-esteem via collective self-esteem (β = -0.27, *p* < 0.001; 95% CI [-0.33, -0.21]; partial mediation: 64.29% of the total effect). The indirect path from collective self-esteem to SWB via personal self-esteem also was significant (β = 0.31, *p* < 0.001; 95% CI [0.21, 0.40]; partial mediation: 63.27% of the total effect).

**Table 3:** Bootstrapping results of the indirect effects in the final model.

Model paths	Estimated	95% CI
Perceived discrimination → CSE → PSE → SWB	-0.16^∗∗∗^	[-0.21, -0.10]
Perceived discrimination → CSE → SWB	-0.10^∗^	[-0.17, -0.03]
Perceived discrimination → PSE → SWB	-0.09^∗^	[-0.15, -0.03]
Perceived discrimination → CSE → PSE	-0.27^∗∗∗^	[-0.33, -0.21]
CSE → PSE → SWB	0.31^∗∗∗^	[0.21, 0.40]


**N* = 692. CSE, collective self-esteem; PSE, personal self-esteem; SWB, subjective well-being; CI, confidence interval. ^∗^*p* < 0.05, ^∗∗∗^*p* < 0.001.*

## Discussion

Previous research has suggested that Chinese migrant children’s experiences of discrimination based on Hukou are one of the most important explanations for their maladjustment (Lin et al., 2009; Shen, 2009). Therefore, there is a compelling need to investigate why perceived discrimination is associated with lower levels of SWB. Expanding on previous research, the goal of this study was to query a model that tested whether or not personal and collective self-esteem mediated the association between perceived discrimination and SWB among Chinese rural-to-urban migrant adolescents. By including both intrapsychic (personal self-esteem) and social–cognitive (collective self-esteem) variables, we hoped to achieve a more comprehensive examination of the relationships among perceived discrimination, personal self-esteem and collective self-esteem, and SWB in Chinese rural-to-urban migrant adolescents.

Low status individuals are not merely passive victims but frequently are able to protect their self-esteem from discrimination through a variety of strategies (Crocker and Major, 1989). Yet the negative contributions of perceived discrimination on self-esteem among low status groups have been documented (Cassidy et al., 2004; Shen, 2009). This study evidenced supported for our main hypothesis; perceived discrimination contributes to lower levels of collective and personal self-esteem, which in turn are associated with lower levels of SWB. Specifically, mediation analysis revealed that self-esteem partially mediated the relation between perceived discrimination and Chinese migrant adolescents’ reported SWB. Prior studies also have suggested that impaired self-concept, in relation to experiences both as an individual and as a group member, is an important outcome of expected and experienced rejection, and may mediate their relationships with negative outcomes (i.e., anxiety and depression) in ethnic minority young people (Cassidy et al., 2004). Our finding that collective and personal self-esteem are mediators between discrimination experiences and SWB—one of the important indicators of positive developmental outcomes—is consistent with this line of research. This indicates that the previously reported mediating role of collective and personal self-esteem (Cassidy et al., 2004) is robust with different low-status groups (i.e., Chinese migrant adolescents) and generates a range of developmental outcomes (i.e., SWB). As a motivational–affective system, self-esteem functions to continuously monitor a person’s social environment for signs of rejection and acceptance (Leary and Baumeister, 2000). Chinese migrant adolescents that believed that they were treated negatively because of Hukou could be particularly sensitive to a lack of social acceptance and exclusion, which could negatively related to their collective and personal self-esteem and further linked with lower levels of SWB.

A resilience framework (Masten and Coatsworth, 1998) posits that aspects of positive self-concept (e.g., collective and personal self-esteem) may minimize the negative connections of risks associated with perceived discrimination. The results of the present study demonstrated that collective and personal self-esteem may predict low level of risk, because they were negatively associated with perceived discrimination, yet positively associated with SWB. Our findings highlight the significant role played by collective and personal self-esteem. They could represent possible targets for intervention among Chinese migrant adolescents.

The study’s findings indicated that the path of perceived discrimination → collective self-esteem → personal self-esteem → SWB was significant. Perceived discrimination was negatively related with Chinese migrant adolescents’ feelings of self-worth and self-respect related to their self-concept as members of the migrant group. Moreover, an undermined collective self-esteem predicted their low sense of personal worth, and, subsequently, their low sense of individual self-worth was connected with a decreased SWB. That is, collective self-esteem was a mediator between perceived discrimination and personal self-esteem while personal self-esteem partially mediated the relationship between collective self-esteem and SWB. Moreover, we also established a reverse model (alternative model 5) to test the complicated relationship among the variables, which provided evidence that SWB may mediate the relationship between perceived discrimination and self-esteem. Results showed that the hypothesized model fit the data well, and reverse model also fit well. A cross-sectional study offered no means by which to differentiate between the hypothesized model and reverse model, and prevented us from drawing causality among study variables. Thus, we do not preclude other potential mediating relations among the study variables and other models remain plausible as alternatives. Future research exploring such relations adopting longitudinal study will contribute to further understanding potential and causal relations among them.

The findings were consistent with the arguments made by Verkuyten and Thijs (2006), who observed that ethnic self-esteem (collective self-esteem) mediated the relationship between ethnic peer discrimination and global self-worth (personal self-esteem) among Turkish, Moroccan, Surinamese, and Dutch young adolescents (aged 10–13) living in the Netherlands (Verkuyten and Thijs, 2006). Our finding was also consistent with existing research that suggested that the relationship between collective self-esteem and SWB was mediated by personal self-esteem (Simsek, 2011). As articulated by Verkuyten and Thijs (2006), discrimination was related to a part of the self; being treated negatively based on one’s ethnic identity (in this instance, perceived Hukou discrimination) had a negative prediction on collective self-esteem and consequently on personal self-worth. Moreover, according to Simsek (2011), personal self-esteem represented a crucial underlying variable that partially explained the relationship between collective self-esteem and SWB. Our findings expanded on previous research by examining the combined roles of collective and personal self-esteem among Chinese migrant adolescents, and revealed a more complicated and nuanced relationship among perceived discrimination, collective self-esteem, personal self-esteem, and SWB. Nevertheless, it is important to note that collective and personal self-esteem only partially mediated the negative association between perceived discrimination and SWB (i.e., 29.09% of the total effect was mediated by collective and personal self-esteem), and that a significant direct link remained between perceived discrimination and SWB. Thus, it will be important to examine other potential mediators that could buffer the relations (e.g., coping skills, social support, and parent–child relationship).

Several limitations of this study merit attention. First, we tried to take collective self-esteem scale as a whole and explore the overall meditational roles of collective self-esteem among perceived discrimination, personal self-esteem, and subjective well-being. In fact, exploring separate roles of each subscales of collective self-esteem scale were also a valuable question which deserves our continued attention in the future studies. Second, data was collected from one province of China, and a convenience sample was used. Future research could benefit from the inclusion of a control group of non-migrant children that could serve to establish baseline levels of self-esteem and subjective well-being as well as provide a basis for comparison. The study’s limitations may limit the ability to make any generalizations from of the study. However, the basic background characteristics of our participants are typical of the disadvantaged group of rural-to-urban migrant adolescents in China (Shen, 2009), which supports the validity of the results obtained. Despite this, their replication with randomly selected samples across the country, including both migrant and non-migrant children, is needed to confirm our conclusions. Third, we used only self-report data, which may have inflated the relationship among variables. Although adolescents are assumed to be able to reliably report their perceptions of discrimination, personal and collective self-esteem, and SWB (Verkuyten and Thijs, 2006; Umaña-Taylor and Updegraff, 2007), it would be useful to employ multiple methods and rating systems in future research; doing so may improve the quality of the response data and thereby the validity of the research findings.

Despite these limitations, this is the first study, to our knowledge, to examine the mediating roles of both collective and personal self-esteem on the relationship between perceived discrimination and SWB in Chinese migrant adolescents. This study advances our understanding of the complicated mechanism through which perceived discrimination is linked with SWB and provides insight to prevention efforts to promote SWB in these vulnerable adolescents. The findings identify personal and collective self-esteem as possible targets for interventions aimed at improving Chinese migrant adolescents’ SWB and counteracting the negative relationships between experienced discrimination and SWB. Therefore, we recommend that parents and teachers encourage and nurture migrant adolescents’ self-esteem, and that school psychologists include more programs that develop self-esteem. Collective self-esteem may be improved through social support from fellow migrant group members. In addition, we also recommend that counselors and teachers help migrant adolescents to positively redefine the “migrant group” for themselves, and provide some lived examples of valuing migrant workers’ experiences and contributions to society, thereby improving their collective self-esteem, which in turn should predict personal self-esteem and SWB levels. Finally, as we found that a direct link from perceived discrimination to SWB remained even after controlling for the contributions of perceived discrimination on personal and collective self-esteem, future work should focus on the role that the central government may play in the prevention and reduction of discrimination against migrant children through changes in the urban-rural household registration system and social welfare policies. It is also important to create non-discriminatory social climates to ensure the positive development of migrant children for local government where migrant children reside.

## Ethics Statement

This study was carried out in accordance with the recommendations of the Research Ethics Committee of Beijing Normal University with written informed consent from all subjects. All subjects gave written informed consent in accordance with the Declaration of Helsinki. The protocol was approved by the Research Ethics Committee of Beijing Normal University.

## Author Contributions

XL conceived and designed the study and supervised the collection of data. XJ and XL analyzed and interpreted the data, and produced the drafting of the manuscripts. BS supervised all steps in the study.

## Conflict of Interest Statement

The authors declare that the research was conducted in the absence of any commercial or financial relationships that could be construed as a potential conflict of interest.

## Footnotes

1 Group membership of migrant children is based on their current place of residence. Although migrant children who obtained rural Hukou have the opportunity to enter primary school and junior high school for study in the city where they currently reside, they must return to rural areas for the senior high school entrance examination and national college entrance exam, which are subject to Hukou restrictions. If those children return to rural residence for school, they are no longer considered to be migrant children. Therefore, in this sense, group membership of migrant children is transitory.

2 In accordance with reforms proposed for the national college entrance examination reform, the Chinese government will enact measures that allow children of migrant workers to take the exam at places where they currently reside. Migrant children will no longer have to return to their place of birth to take the high-school entrance examination in the future.
